# Validation of the cobas 6800 human papillomavirus test in primary cervical screening

**DOI:** 10.1371/journal.pone.0247291

**Published:** 2021-02-19

**Authors:** Karin Sundström, Helena Lamin, Joakim Dillner

**Affiliations:** Department of Laboratory Medicine, Center for Cervical Cancer Prevention, Karolinska Institutet and Karolinska University Laboratory, Karolinska University Hospital, Stockholm, Sweden; Azienda Ospedaliero Universitaria Ospedali Riuniti di Ancona Umberto I G M Lancisi G Salesi, ITALY

## Abstract

Evaluation of Human Papillomavirus (HPV) testing systems suitable for large-scale organized cervical screening programs is required. We evaluated the cobas 6800 HPV test system for detection of cervical intraepithelial neoplasia grade 3 or worse (CIN3+) when nested in an organized primary HPV screening program, using the cobas 4800 test as comparator. The Karolinska University Hospital Cervical Cytology Biobank, containing frozen cervical samples from >700,000 women participating in organized cervical screening, was linked to the Swedish national cervical screening registry to identify 470 stored cervical samples taken <180 days before histopathological diagnosis of CIN3+. Two controls per case, with no abnormal results for 2 screening rounds, matched for age and sampling time were also retrieved. Aliquots from 1406 women were retrieved and re-tested on the cobas 4800 system and tested on the cobas 6800 system. There was high reproducibility between the original cobas 4800 HPV test results, and the cobas 4800 HPV re-testing performed on the samples retrieved from biobank storage. 462/464 biobanked samples from women with CIN3+ tested HPV-positive on the cobas 6800 system, corresponding to a relative sensitivity of 99.6%. 925/932 biobanked samples from control women tested HPV-negative on the cobas 6800 platform, corresponding to a relative specificity of 99.2%. By conventional criteria, the cobas 6800 was non-inferior both regarding relative sensitivity of >90% (non-inferiority p-value <0.0001) and relative specificity of >98% (non-inferiority p-value 0.006). We conclude that the cobas 6800 HPV test system had similar, high performance as the cobas 4800 such, when evaluated using cervical samples taken before CIN3+ in a real-life primary HPV screening program.

## Introduction

Organized cervical screening programs reduce the incidence of cervical cancer. However, it is still the third or fourth most common cancer among women worldwide (IARC). Persistent infection with oncogenic types of human papillomavirus (HPV) is the major cause of cervical cancer [1]. Oncogenic types of *Human papillomavirus* belongs to the *Papillomaviridae* family, genus *Alphapapillomavirus*. Major randomized controlled trials have found that HPV-based screening is superior over cytology screening to prevent cervical cancer [2,3]. Today, primary HPV-based cervical screening is globally recommended and implemented in numerous countries, and there is therefore a need for jointly agreed standard criteria for structured evaluation of new high throughput HPV testing systems validated in the population-based screening setting [4]. These criteria focus on the need to show clinical utility of the new system to detect not oncogenic HPV per se, or low-grade lesions, but true cervical cancer precursor lesions in the form of cervical intraepithelial neoplasia grade 2 or worse (CIN2+) and CIN grade 3 or worse (CIN3+). This since it is key that show that assays are capable of distinguishing HPV infections associated with clinically relevant lesions, and not just transient HPV infections [4].

The cobas 4800 system is an automated system that has for a number of years been a reference standard for HPV screening programs. For example, the organized cervical screening program in Stockholm installed cobas 4800 in 2011. The system has 97% sensitivity for CIN3+ [5] and has been successfully deployed for primary HPV-based screening [6]. However, the system is medium throughput (384 samples/day). The cobas 6800 is a high throughput system (>1500 samples/day) that is widely in use in large-scale laboratories performing molecular testing for viruses.

We here investigate whether the new cobas 6800 system for HPV PCR analyses has appropriate sensitivity and specificity for prediction of histologically confirmed cervical intraepithelial neoplasia grade 3 or worse (CIN3+) in a population-based longitudinal cohort study, using the already established cobas 4800 system as the comparator assay. We focus on CIN3+ rather than CIN2+ since it is a more reproducible diagnosis and is equivalent to carcinoma in situ; thus placing it higher on the scale of clinical utility.

## Materials and methods

### Setting

By a 2015 decision of the Swedish National Board of Health and Welfare (a Swedish government agency), the Swedish organized cervical screening program must invite all resident women to screening every 3 years between the ages of 23 and 49 years, and every 7 years between the ages of 50 and 70. The sample taken is liquid-based and the primary screening test is cytology for ages 23–29 and HPV for ages 30–70. In the capital region of Sweden (about 23% of the population of the country), the Center for Cervical Cancer Prevention (CCCP) performs all the HPV testing within the program and all residual ThinPrep liquid-based cytology (LBC) cervical samples are stored at -25°C after testing [7]. CCCP is also managing the Swedish National Cervical Screening Registry (Swedish abbreviation NKCx, www.nkcx.se/index_e.htm). Both the screening program, the biobank and the NKCx store identifiable data, possible to link via the unique Swedish personal identification number.

The HPV screening test used for population-based cervical screening (as well as for any clinical HPV testing) at CCCP is determined by purchasing. The cobas 4800 system (Roche) won the public tender in 2011 and again in 2016.

### Identification of cases and controls

**Fig 1:**
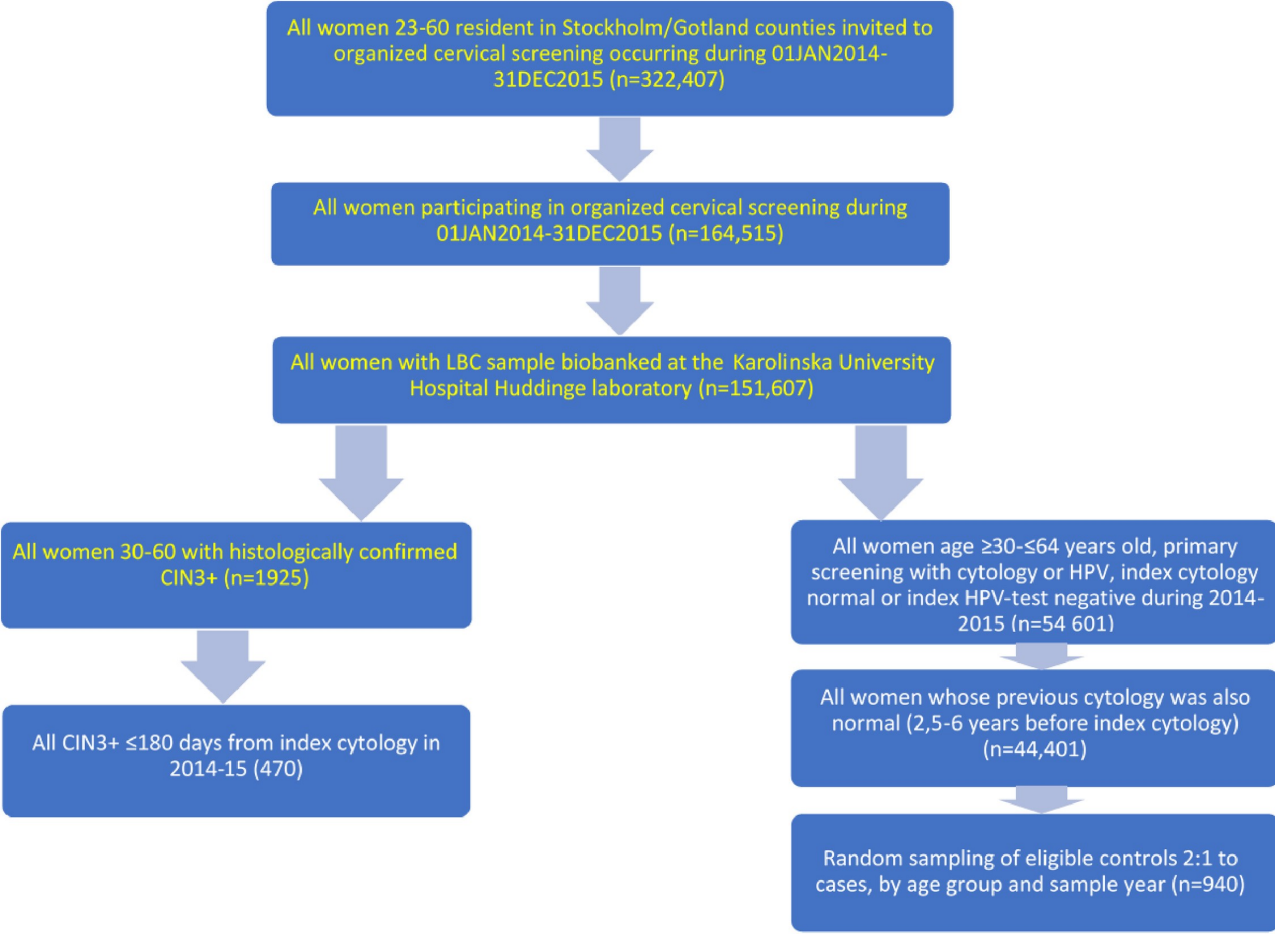
Flow chart of the study participants. LBC = liquid-based cytology; CIN3+ = cervical intraepithelial neoplasia grade 3 or worse (including adenocarcinoma in situ/AIS and invasive cervical cancer).

This study was performed entirely by using biobanked LBC samples from women participating in the primary organized cervical screening program in the Stockholm-Gotland region. Samples deriving from clinically indicated (i.e. symptom-driven) testing were not included. We restricted the study to samples biobanked during 2014 and 2015. Since 2014, HPV-based screening was widely used in the organized program. The database with eligible 151,607 samples was linked to NKCx to identify all screening-derived LBC samples from women who had a CIN3+ diagnosis in histopathology (n = 1925). From these 1925 women, we identified and included all who had a CIN3+ diagnosis within 180 days from the index LBC, a total of 470 samples from 470 women. As potential controls, we identified all women participating in screening who had an index LBC which was negative (either cytologically normal or HPV-negative) and who had not been diagnosed with any previous cervical disease in histopathology (n = 54,601). Among these, 44,401 women had been either HPV-negative or had a normal cytology also in the previous screening round. Among the 44,401 eligible control women, we selected 2 controls per case, matched by birth year and calendar year of taking the LBC samples, a total of 940 samples from 940 healthy women (Fig 1).

Among the identified samples, biobank aliquots from 468/470 cases (99.6%) and 938/940 controls (99.8%) could be retrieved. The sample aliquots were first tested on the cobas 4800 system, for comparison with the original results obtained when testing the fresh samples in the organized screening program, and then on the cobas 6800 system.

The comparison between biobanked and fresh samples had a preassigned performance criterion that the concordance between the cobas 4800 testing of the fresh sample and the biobanked sample should be >95%.

### Laboratory analyses

The cobas platforms are based on the real-time polymerase chain reaction (PCR) method, and both assays can detect 14 HPV types (high-risk types (hr) HPV16/18/31/33/35/39/45/51/52/56/58/59 and HPV66 and 68). Positivity for HPV16 and/or HPV18 are reported in two separate type-specific fluorescence channels, while infection with one or more of the other12 types is reported in a third fluorescence channel as a single result, “other hrHPV positive”. Staff from Roche trained the laboratory personnel responsible for this study locally. Both instruments were already available at the local hospital site.

### Statistical analyses

The McNemar test with continuity correction for paired categorical data was applied to determine the p-value associated with comparison of test results between assays. In this approach, a p-value >0.05 and odds ratio (OR) with 95% confidence interval (CI) containing 1.00 signifies that there is no statistically significant observed difference in performance between the new test and the comparator test.

Furthermore, as recommended by the consensus article by Meijer et al, we performed a non-inferiority test for the comparison of cobas 6800 sensitivity relative to cobas 4800, as previously described [4]. In this test, the null hypothesis is that the relative sensitivity is <90% and that the relative test specificity is less than 98%. If the p-value is below 0.05, there is statistical evidence that the new assay is non-inferior.

### Ethical approval

The study was approved by the Regional Ethical Review Board of Stockholm (approval numbers 2017/816-32 and 2018/368-32), which determined that study-specific informed consent from the study participants was not required due to the reuse of pseudonymized samples collected through population-based screening where women have already consented to have their sample available for ethically approved research studies, unless the woman chooses to opt out of such use. The written invitations for screening include information about the registry and the biobank, including information on how to opt out of the system.

### Data citation

The sampling frame data-set used for this manuscript was accessed through the Swedish National Cervical Screening Registry (NKCx), http://www.nkcx.se/research_e.htm.

## Results

### Sensitivity

In the original cobas 4800 testing in the organized screening program, 466/468 case women (99.6%) had been high-risk HPV (hrHPV) positive. In the repeat cobas 4800 testing of the biobanked residual samples, two samples had an invalid result and were therefore excluded. Of the remaining samples, 462/464 case women (99.6%) were hrHPV positive. 227 women were HPV16 positive in the original testing; 223/227 (98.2%) were HPV16 positive also in the re-testing with cobas 4800 (Table 1). No women who were originally HPV16 negative re-tested as HPV16 positive. Similarly, 51 women were HPV18 positive in the original testing; 49 (96.1%) were HPV18 positive also in the re-testing. Again, no woman originally negative for HPV18 re-tested as HPV18 positive. Lastly, 294 women were other hrHPV positive in the original testing, 286 (97.3%) of which re-tested as other hrHPV positive. Some women who were originally other hrHPV negative re-tested as other hrHPV positive: 8/170 women (4.7%) (Table 1). These results were above the prespecified minimum performance criterion that concordance between testing of fresh sample and of biobanked sample should be >95% and testing was therefore continued with testing of the biobanked samples with cobas 6800.

**Table 1:** Distribution of HPV positive and negative results in the original cobas4800 test, and the re-test cobas4800 result, respectively.

	Test result						
	HPV16 positive	HPV16 negative	HPV18 positive	HPV18 negative	Other hrHPV positive	Other hrHPV negative	Total**
**Original cobas4800**	227	236	51	412	294	170	464
**Re-testing cobas4800**	223	236	49	412	294*	162	464

*286/294 re-tested as other hrHPV positive, plus an additional 8/170 originally other hrHPV negative samples.

**Columns may sum to more than 464 test results, due to some samples being positive for more than one category.

In the cobas 6800 de novo testing of the biobanked samples, the same two samples that were invalid on the cobas 4800 re-testing also tested invalid on the cobas 6800 platform and were thus excluded. 462/464 case women (99.6%) were positive in the cobas 6800 testing (Table 2), which was the same proportion as in the cobas 4800 re-testing of the same archival samples (p = 0.7237, OR 1.00 (95% CI 0.19–5.4)). The p-value from non-inferiority testing was <0.0001, indicating that the sensitivity of the cobas 6800 testing was non-inferior to the original cobas 4800 testing (Table 2).

**Table 2:** Comparison of cobas 4800 and cobas 6800 sensitivity in the 464 pairs of valid samples from women with CIN3+.

	cobas4800+	cobas4800-	Total	*P*_ni_
cobas 6800+	456	4	460	
cobas 6800-	4	0	4	
	460	4	464	<0.0001

*p*_ni_ = p-value from non-inferiority testing. A P-value<0.05 signifies that the null hypothesis of inferiority is rejected and that the sensitivity of the new cobas 6800 testing is non-inferior to the original cobas 4800 result.

### Specificity

By design, none out of the 938 control women were hrHPV positive in the original cobas 4800 analyses performed in the screening program.

Two samples were invalid on the cobas 4800 re-testing and so were excluded. Upon this re-testing, 936 samples re-tested as hrHPV negative, but 3/936 samples (0.3%) were positive in the cobas 4800 re-testing of the same samples after retrieval from biobank storage (all re-tested positive for “other hrHPV”, similar to the sensitivity arm). In the cobas 6800 de novo testing of the biobanked samples, an additional 4 samples were invalid and excluded, for a total of 932 samples. 925 tested as hrHPV negative on cobas6800 (yielding a relative specificity of 925/932 = 0.992), but 8/932 samples (0.9%) from control women were positive in the cobas 6800 analyses of the same biobanked samples (p = 0.2278 compared to the proportion in the cobas 4800 re-testing, i.e. similar. The OR was 0.35, 95% CI 0.06–1.56)). The p-value from non-inferiority test was p = 0.006, indicating that the specificity of cobas 6800 was non-inferior compared to the cobas 4800 (Table 3).

**Table 3:** Comparison of cobas 4800 and cobas 6800 specificity in the 933 pairs of valid samples from control women without CIN3+.

	cobas4800+	cobas4800-	Total	*P*_ni_
cobas6800+	0	8	8	
cobas6800-	3	921	924	
	3	929	932	0.006

*p*_ni_ = p-value from non-inferiority testing. A P-value<0.05 signifies that the null hypothesis of inferiority is rejected and that the specificity of the new cobas 6800 testing is non-inferior to the original cobas 4800 result.

### Concordance

Direct comparison of the cobas 4800 and cobas 6800 results in the same archival samples showed a very high concordance (97% of samples had identical results on both platforms). Variability regarding a test result classified as “invalid” was found in 9 samples. Four case samples were hrHPV positive in cobas 4800 but hrHPV negative in cobas 6800, and four other case samples were hrHPV negative in cobas 4800 but hrHPV positive in cobas 6800. Three control samples were hrHPV positive in cobas 4800 but negative in cobas 6800 and eight samples were hrHPV positive in cobas 6800 but hrHPV negative in cobas 4800.

## Discussion

We found that in the setting of primary HPV-based screening in a population-based organized program, cobas 6800 had an overall sensitivity for subsequent histopathology-verified CIN3+ of 99,1%, which was identical to the sensitivity of the cobas 4800 HPV test system. The specificity was found to be likewise highly similar (relative specificity 99,1%). The relative sensitivity and specificity performance of cobas 6800 was non-inferior to cobas 4800 by the thresholds deemed adequate to detect clinically relevant cervical lesions [4].

The strengths of this study include the use of a comprehensive cohort which systematically collects and stores residuals from all samples in primary cervical screening in the capital region of Sweden. Combined with a comprehensive national cervical screening registry catching all subsequent histopathological samples in the entire country, we had excellent information on exposure and outcomes in the population at risk. Thus, we could perform a new test system evaluation in a maximally generalizable setting (population-based screening) with a maximally reliable study design (longitudinal cohort study with complete and nationwide registry-based follow-up).

The limitations of this study include that the comparison was based on archival samples, rather than on fresh samples taken in the screening program. We did note 1–4.7% units increased detection of other hrHPV upon re-testing in the cobas 4800, and the de novo testing in the cobas 6800, respectively, compared to the original testing performed in the screening program. This finding was similar between the two assays, and no similar increased detection of HPV16 and/or HPV18 DNA was observed. As these would be low-level infections with less oncogenic types than HPV16/18, the observation is not likely to be of clinical significance.

The overall comparability between prospectively tested fresh samples and testing of archival samples was evaluated and found to be well above the prespecified performance criteria. Also, the fact that the cobas 6800 testing was done only after case ascertainment means that these hrHPV results cannot have affected the original definition of CIN3+ case outcomes diagnosed in 2014–2015. Thus, misclassification deriving from the use of archived samples would be non-differential regarding case-control status and cannot affect the validity or generalizability of our observations.

As cobas 4800 had been used for original primary screening in the screening program, it is effectively a “gold standard” test where comparison of a new test cannot possibly result in *better* performance than the comparator test. However, the results of the new test were so close to the results of the gold standard test that it can safely be concluded that it has similar performance.

Recently, in a study separate from ours, the cobas 6800 was clinically evaluated according to the Meijer criteria. Similar to our results, they observed high agreement (>98%) for the two assays, and that clinical sensitivity and specificity met the non-inferiority criteria defined to consider an HPV assay as suitable for large-scale laboratory use [8]. Similar conclusions were reached by another validation study in the Australian population-based setting [9], where a relative specificity of 98.9% was observed.

We conclude that the cobas 6800 HPV test system had a similar, high performance as the cobas 4800 HPV test system when evaluated using cervical samples taken before CIN3+ in a real-life primary HPV screening program. There are now three studies in distinct cohorts, which show similar results on the clinical validity of the system.
